# Biomarker analysis of on-treatment and at progression biopsies from BRIM7 - a phase 1B trial of combined vemurafenib and cobimetinib treatment in BRAF V600 mutated melanoma

**DOI:** 10.1186/1479-5876-12-S1-O12

**Published:** 2014-05-06

**Authors:** Yibing Yan, Grant McArthur, Omid Hamid, Igor Puzanov, Rene Gonzalez, Thomas Gajewski, Yulei Wang, Matthew Wongchenko, Nicholas Choong, Antoni Ribas

**Affiliations:** 1 Genentech, South San Francisco, CA, USA; 2 Peter MacCallum Cancer Centre, East Melbourne, Victoria, Australia; 3 The Angeles Clinic and Research Institute, Los Angeles, CA, USA; 4 Vanderbilt-Ingram Cancer Center, Nashville, TN, USA; 5 University of Colorado Comprehensive Cancer Center, Aurora, CO, USA; 6 University of Chicago, Chicago, USA; 7 The Jonsson Comprehensive Cancer Center at University of California, Los Angeles, CA, USA

## Background

Combined BRAF and MEK inhibition may delay the onset of resistance compared with BRAF inhibition alone. The safety and tolerability of the BRAF and MEK inhibitors, vemurafenib and cobimetinib were evaluated in a phase 1B trial, BRIM7 (NCT01271803). Modulation of signaling pathways, transcriptional outputs and T-cell dynamics were assessed in tumor samples obtained from BRIM7.

## Materials and methods

Tumor tissues were collected at baseline prior to treatment, on-treatment at cycle 1 day 14 (C1D14) and at disease progression (PD). Modulation of the MAPK and PI3K pathways, cell proliferation, and CD8^+^ T-cells in tumor biopsies were assessed by immunohistochemistry. Changes in transcription profiles upon treatment were measured by qRT-PCR using the Fluidigm/Nanostring platforms.

## Results

133 tumor samples have been collected representing 82 unique patients. There are six paired biopsies (baseline and C1D14) from the same patients, and seven evaluable PD samples. In the six paired biopsies (baseline and C1D14), robust inhibition of the MAPK pathway was observed in tumors following vemurafenib + cobimetinib treatment as measured by reduction of pERK levels compared to baseline (mean H-score inhibition 88 +/- 12%). This was observed in samples from BRAF inhibitor-naïve patients (n=2) and also from patients who had progressed while on vemurafenib (n=4). The inhibition of pERK correlated with the reduction of the proliferation marker Ki67 in C1D14 tumor samples (77 +/- 10%), while inhibition of the PI3K pathway marker pS6 showed greater variability (68 +/- 28%). At PD, varying levels of MAPK pathway activity and moderate-to-high expression of Ki67 were seen in tumors. Ongoing analyses of combination treatment effects on the MAPK pathway, immune regulatory gene transcription and CD8+T-cell status will be presented.

## Conclusions

The combination of vemurafenib + cobimetinib results in MAPK pathway inhibition in both BRAF inhibitor naïve patients and patients who have progressed on vemurafenib. Increased levels of pERK and Ki67 at PD suggest the renewal of proliferation as tumors may have escaped the inhibitory effects of the combination therapy. Effects of the combined treatment on the MAPK pathway and immune gene signatures will also be discussed.

